# Lymph Node Ratio Enhances Predictive Value for Treatment Outcomes in Patients with Non-Small Cell Lung Cancer Undergoing Surgery: A Retrospective Cohort Study

**DOI:** 10.7150/jca.90525

**Published:** 2024-01-01

**Authors:** Shou-Feng Wang, Nai-Quan Mao, Jiang-Qiong Huang, Xin-Bin Pan

**Affiliations:** 1 Department of Thoracic surgery, Guangxi Medical University Cancer Hospital, Nanning, Guangxi 530021, P.R. China.; 2 Department of Radiation Oncology, Guangxi Medical University Cancer Hospital, Nanning, Guangxi 530021, P.R. China.

**Keywords:** non-small cell lung cancer, surgery, lymph node ratio, survival.

## Abstract

***Purpose:*** To compare the prognostic value of lymph node ratio (LNR) and pN in patients with non-small cell lung cancer (NSCLC) undergoing surgery.

***Materials and methods:*** NSCLC patients were investigated between 2004 and 2015 from the Surveillance, Epidemiology, and End Results databases. The X-tile software was used to determine LNR cut-off values. Kaplan-Meier analysis was employed to assess cancer-specific survival (CSS) and overall survival (OS).

***Results:*** The identified cut-off values of LNR were 0.19 and 0.73. Median CSS for LNR1 (LNR < 0.19), LNR2 (0.19 ≤ LNR ≤ 0.73), and LNR3 (LNR > 0.73) were 71, 41, and 17 months. Both LNR2 (HR = 1.46, 95% CI: 1.36-1.57; P < 0.001) and LNR3 (HR = 2.85, 95% CI: 2.58-3.15; P < 0.001) demonstrated poorer median CSS compared to LNR1. Similarly, median OS for LNR1, LNR2, and LNR3 were 50, 35, and 16 months. LNR2 (HR = 1.36, 95% CI: 1.27-1.45; P < 0.001) and LNR3 (HR = 2.60, 95% CI: 2.37-2.85; P < 0.001) exhibited worse median OS compared to LNR1. A revised pN (r-pN) classification incorporating LNR and pN demonstrated superior penalized goodness-of-fit and discriminative ability in predicting CSS and OS compared to both LNR and pN.

***Conclusion:*** LNR outperformed pN in predicting CSS and OS in NSCLC patients undergoing surgery, potentially leading to more precise adjuvant treatment decisions.

## Introduction

Lung cancer is a major contributor to global cancer-related mortality, accounting for 18.0% of such deaths.^1^, ^2^ Non-small cell lung cancer (NSCLC) comprises approximately 85% of these cases.^3^ The current standard of care for resectable NSCLC involves radical resection and lymph node dissection.^4^ The pN stage, determined by lymph node sampling after surgery, is a major prognostic factor for survival.^5^ Consequently, the precision of lymph node sampling is a critical aspect of surgical management, and it plays a pivotal role in the decision regarding adjuvant therapies.

The National Comprehensive Cancer Network (NCCN) guidelines recommend the sampling of lymph node stations with one or more nodes from all mediastinal stations. However, the minimum number of lymph nodes to be examined remains a point of debate. The International Association for the Study of Lung Cancer (IASLC) and the European Society of Thoracic Surgery (ESTS) recommend a minimum of 6 examined lymph nodes.^6^, ^7^ In contrast, the Chinese Journal of Oncology suggests a minimum of 12 examined lymph node.^8^

The impact of examined lymph nodes on prognosis is a subject of ongoing discussion. Studies have demonstrated a connection between the number of examined lymph nodes and patient survival, with a higher count associated with improved prognosis.^9^, ^10^ It has been suggested that 10 examined lymph nodes represent an adequate cut-off value for dissection, beyond which there is no further improvement in prognosis.^10^ However, others propose 16 examined lymph nodes as the threshold for assessing the quality of lymph node examination and postoperative prognostic stratification in NSCLC patients who undergo surgery.^11^ Furthermore, the effectiveness of examined lymph nodes as a representation of pN stage can be influenced by surgical quality.^12^

The lymph node ratio (LNR), defined as the ratio of pathologically metastatic lymph nodes to the total number of harvested examined lymph nodes, has been used as a prognostic factor in various cancers, including colorectal cancer^13^, breast cancer^14^, and gastric cancer^15^. In this study, we utilized data from the Surveillance, Epidemiology, and End Results (SEER) databases to compare the prognostic value of LNR with the conventional pN stage classification. Additionally, our objective is to propose a revised pN (r-pN) classification based on LNR and pN stages.

## Materials and methods

### Data source

Data for this study were extracted from the SEER databases, which contain de-identified information from population-based cancer registries in the United States. Data extraction was performed using SEER*Stat software version 8.3.6 (www.seer.cancer.gov/seerstat).

### Patient population

The study included NSCLC patients diagnosed between 2004 and 2015 who met specific inclusion criteria: (1) histopathologically confirmed adenocarcinoma or squamous cancer, (2) age ≥ 18 years, (3) initial therapy involving surgery, (4) lymph node examination performed, (5) definite tumor-node-metastasis (TNM) staging according to the 7th edition of the American Joint Committee on Cancer (AJCC) staging system, (6) non-M1 and non-N0 stage, and (7) no chemotherapy or radiotherapy.

Patient characteristics, including age, sex, race, primary site, tumor grade, histological types, T stage, N stage, number of resected lymph nodes, and number of positive lymph nodes, were extracted.

### Endpoints

The primary endpoint, cancer-specific survival (CSS), was defined as the time between diagnosis and death attributed to lung cancer as recorded in the SEER database. The secondary endpoint, overall survival (OS), measured the time from diagnosis to death due to any cause within the SEER database.

### Stratification of lymph node ratio

The LNR was calculated as the ratio of pathologically metastatic lymph nodes to the total number of harvested examined lymph nodes. To establish optimal LNR cut-off values, we utilized the X-tile software (http://www.tissuearray.org/rimmlab). X-tile generated plots by categorizing LNR into three groups: low, middle, and high-risk groups. It systematically evaluated all possible divisions of LNR and calculated the associated associations using the log-rank test for survival.

The X-tile software identified the optimal LNR division by selecting the point with the highest χ^2^ value. To assess statistical significance, we applied the cut-point derived from a training set to analyze a separate validation set, employing a standard log-rank test and obtaining P values from a lookup table.

The cut-off values determined by X-tile were 0.19 and 0.73. Based on these values, we defined three LNR categories, referred to as LNR stages: LNR1 (LNR < 0.19), LNR2 (0.19 ≤ LNR ≤ 0.73), and LNR3 (LNR > 0.73). To maintain consistency with the 7th edition of the AJCC staging system, we developed a revised pN (r-pN) classification. This r-pN classification stratified the current pN categories into r-pN categories based on LNR stages.

### Comparison of predictive performance

The predictive performance of pN, LNR, and r-pN stages was assessed using Harrell's concordance index (C-index) and Akaike information criterion (AIC).^16^, ^17^ Harrell's C-index measures the proportion of correctly ordered pairs of patients' predicted survival times among all possible pairs. Higher C-index values indicate better discrimination. The AIC is calculated as (2 × the number of parameters in the model)-(2 × the log maximum likelihood), with lower AIC values indicating better calibration.

### Statistical analysis

Age, categorized based on 60 years, and categorical variables such as sex, race, primary site, tumor grade, histological types, and T stage were compared among N stages, LNR stages, and r-pN stages using the χ2 test or Fisher's exact test.

CSS and OS were compared using Kaplan-Meier methods, with log-rank test statistics applied between pN subgroups. Similar comparisons were made between LNR subgroups. Survival curves for different risk subgroups of the r-pN stages were generated using the Kaplan-Meier method and subjected to pairwise log-rank tests.

Statistical analyses were conducted using SPSS Statistics Version 26.0 software (IBM Co., Armonk, NY, USA) and R software (version 4.2.2). Statistical significance was determined using two-tailed P values below 0.05.

## Results

### Patient selection and characteristics

**Figure 1:**
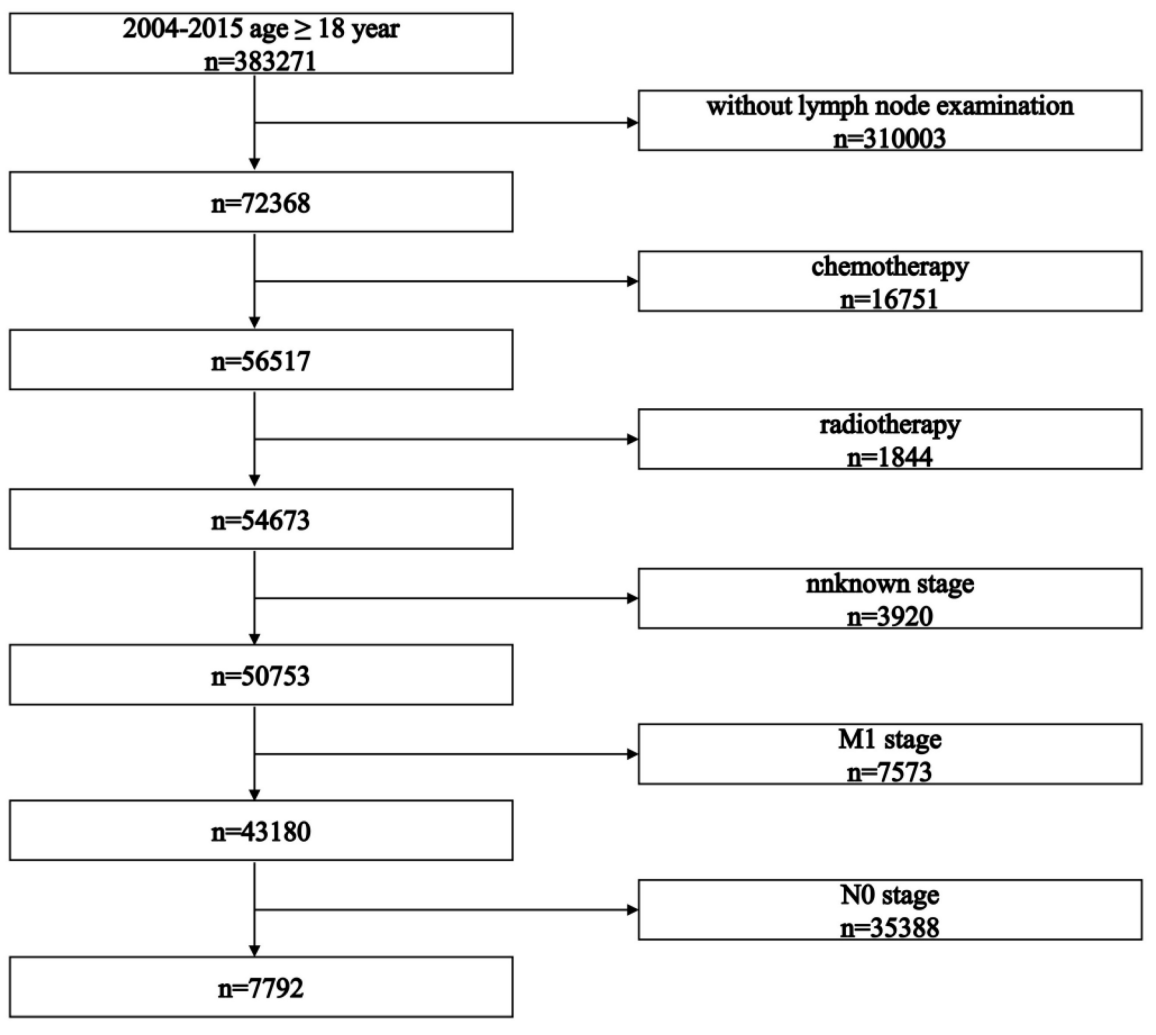
Flowchart illustrating the patient selection process.

The selection process is presented in Figure 1. The study initially identified 383,271 patients. After applying inclusion criteria, 7,792 patients who received surgery as initial treatment were included in this retrospective cohort study. Baseline characteristics of all included patients are summarized in Table 1. Baseline characteristics between LNR stages and pN stages are summarized in Tables 2 and 3, respectively.

### Cancer-specific survival

**Figure 2:**
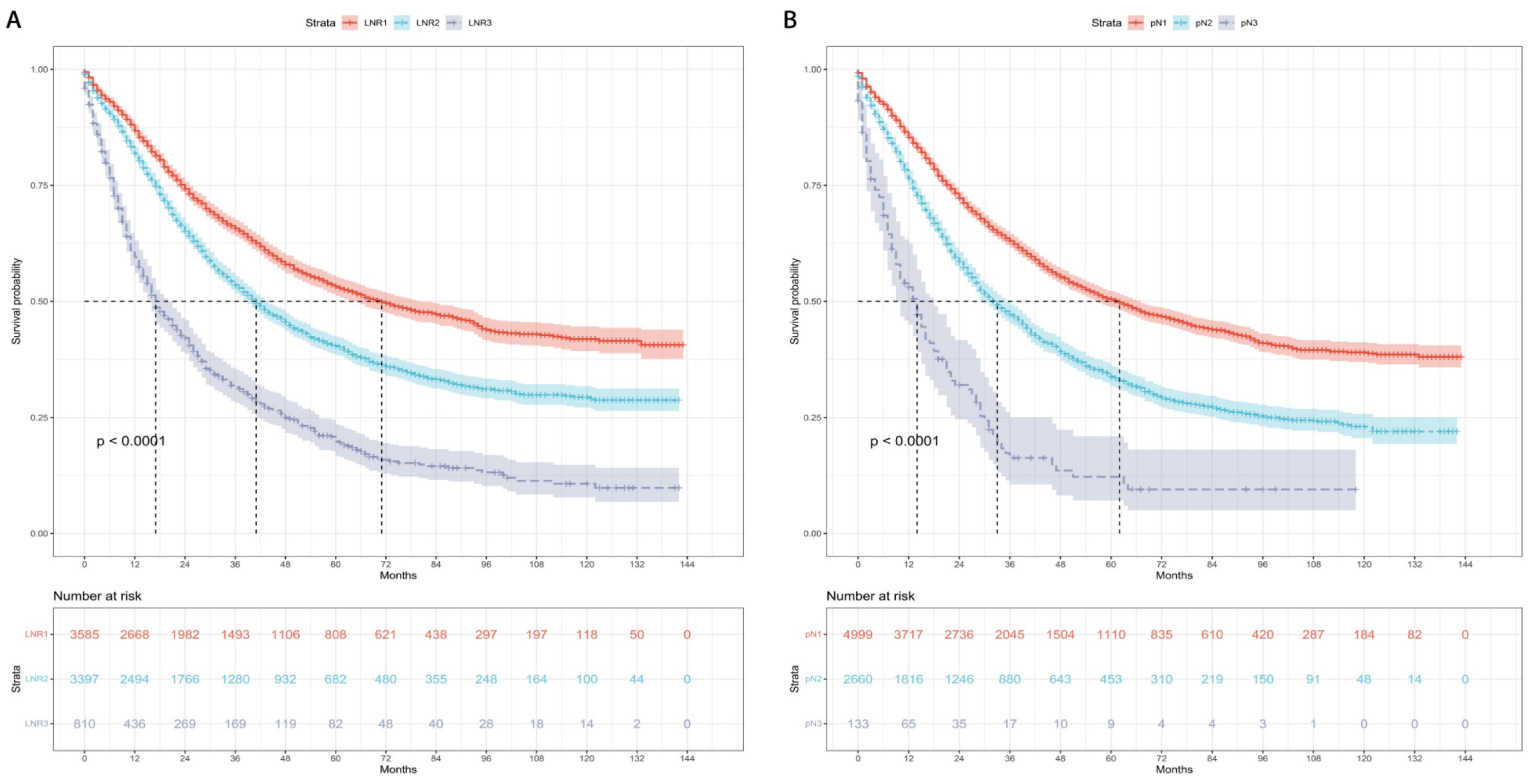
Cause-specific survival based on the LNR and pN stages. (A) LNR stage. (B) pN stage. LNR: lymph node ratio. LNR1: LNR < 0.19, LNR2: 0.19 ≤ LNR ≤ 0.73,

Kaplan-Meier curves and risk tables of CSS for LNR stages are illustrated in Figure 2A. The median CSS rates for patients with LNR1, LNR2, and LNR3 were 71, 41, and 17 months, respectively (Table 4). Pairwise comparisons revealed significantly different median CSS rates. LNR2 (HR = 1.46, 95% CI: 1.36-1.57; P < 0.001) and LNR3 (HR = 2.85, 95% CI: 2.58-3.15; P < 0.001) had worse median CSS compared to LNR1.

Kaplan-Meier curves and risk tables of CSS for pN stages are depicted in Figure 2B. The median CSS rates for patients with pN1, pN2, and pN3 were 62, 33, and 14 months, respectively (Table 4). Significant differences in median CSS rates were observed in pairwise comparisons. pN2 (HR = 1.61, 95% CI: 1.51-1.73; P < 0.001) and pN3 (HR = 3.21, 95% CI: 2.63-3.92; P < 0.001) had worse median CSS compared to pN1.

### Overall survival

**Figure 3:**
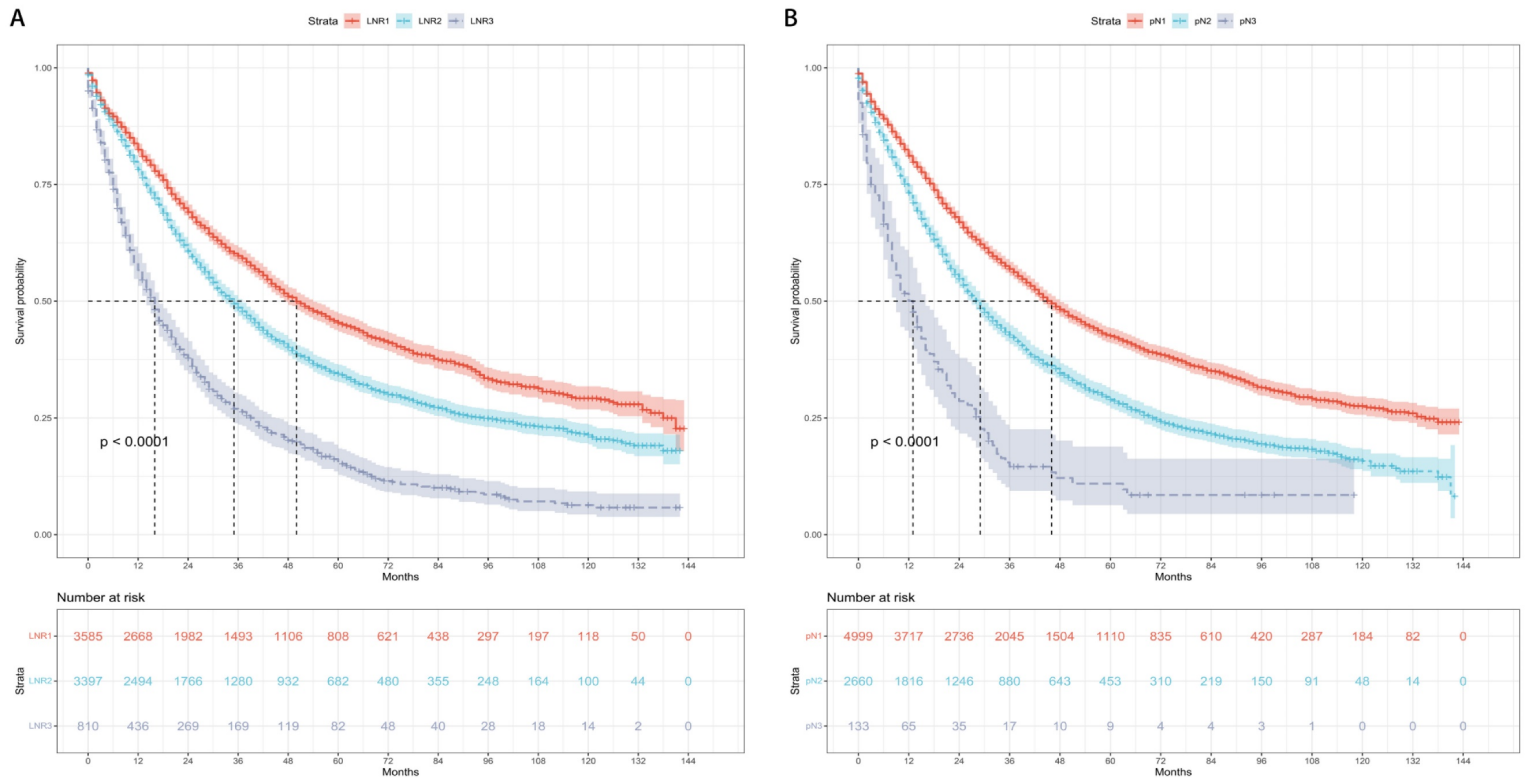
Overall survival based on the LNR and pN stages. (A) LNR stage. (B) pN stage. LNR: lymph node ratio. LNR1: LNR < 0.19, LNR2: 0.19 ≤ LNR ≤ 0.73, LNR3: LNR > 0.73.

Kaplan-Meier curves and risk tables of OS for LNR stages are presented in Figure 3A. The median OS rates for patients with LNR1, LNR2, and LNR3 were 50, 35, and 16 months, respectively (Table 4). Pairwise comparisons revealed significantly different median OS rates. LNR2 (HR = 1.36, 95% CI: 1.27-1.45; P < 0.001) and LNR3 (HR = 2.60, 95% CI: 2.37-2.85; P < 0.001) had worse median OS compared to LNR1.

Kaplan-Meier curves and risk tables of OS for pN stages are shown in Figure 3B. The median OS rates for patients with pN1, pN2, and pN3 were 46, 29, and 13 months, respectively (Table 4). Significant differences in median OS rates were observed in pairwise comparisons. pN2 (HR = 1.50, 95% CI: 1.41-1.60; P < 0.001) and pN3 (HR = 2.81, 95% CI: 2.33-3.41; P < 0.001) had worse median OS compared to pN1.

### Predictive performance

Table 5 presents the AIC and Harrell's C-index, comparing the predictive performance of LNR, pN, and r-pN stages in multivariable proportional hazards models. The LNR stages exhibited better penalized goodness-of-fit (AIC: 59,919 vs. 60,040) and better discriminant ability (Harrell's C-index: 0.648 vs. 0.640) than the pN stages in predicting CSS. Similarly, the LNR stages showed better penalized goodness-of-fit (AIC: 72,957 vs. 73,100) and better discriminant ability (Harrell's C-index: 0.642 vs. 0.633) than the pN stages in predicting OS.

The r-pN stages deed the best predictive performance among LNR stages, pN stages, and r-pN stages. In predicting CSS, the AIC was 59,814, and the Harrell's C-index was 0.656. In predicting OS, the AIC was 72,872, and the Harrell's C-index was 0.648.

**Figure 4:**
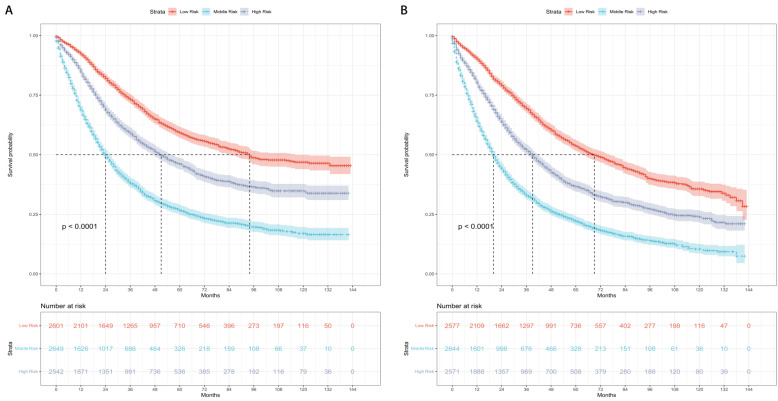
Kaplan-Meier survival curves for the revised pN stages categorized into low-risk, medium-risk, and high-risk subgroups. (A) Cause-specific survival. (B) Overall survival.

The patients were categorized into three risk subgroups (low-risk, medium-risk, and high-risk) according to the r-pN stages. A significant difference in the prognostic classification for predicting CSS and OS was observed through the log-rank test (P < 0.001) (Figure 4). Consequently, the r-pN stages proved to be effective in distinguishing both CSS and OS among the suggested risk subgroups of NSCLC patients.

## Discussion

The TNM staging system is a critical prognostic factor in NSCLC, with N stage being among the most significant, as survival substantially decreases with increasing N stage.^18^ Nevertheless, N stage relies solely on the location and involvement of positive lymph nodes, which may not fully encapsulate the biological heterogeneity present in NSCLC.^19^ Patients sharing the same N stage can still exhibit diverse treatment outcomes, underscoring the need for a more precise classification based on N stage.^20^

Previous studies have highlighted the prognostic importance of examining lymph nodes in NSCLC patients,^9^, ^10^, ^21^, ^22^ recognizing adequate lymphadenectomy as an independent prognostic factor correlated with better outcomes. However, discrepancies in surgical quality among hospitals and surgeons may affect the extent of lymph node examination, potentially limiting its ability to accurately represent the true N stages.^5^, ^12^

The LNR, considering both the number of positive lymph nodes and the number of examined lymph nodes, had emerged as a valuable prognostic factor in NSCLC.^23^-^25^ Our study reaffirmed the LNR's significance as an independent prognostic factor for both CSS and OS. Moreover, the LNR categories (LNR1 [LNR < 0.19], LNR2 [0.19 ≤ LNR ≤ 0.73], and LNR3 [LNR > 0.73]) demonstrated superior discriminatory and predictive abilities for prognosis compared to pN stages. These findings indicated that the LNR is a simple, convenient, and reproducible prognostic factor that can effectively stratify NSCLC patients.

In conclusion, our study emphasizes the prognostic significance of the LNR in NSCLC patients undergoing radical surgery. LNR staging outperforms pN staging in terms of predictive accuracy. Additionally, the r-pN classification, which integrates both LNR and pN stages, holds promise for tailoring more precise adjuvant treatment strategies.

## Figures and Tables

**Table 1:** Description of baseline patient characteristics.

Variables	Overall (N=7792)
Age	
≤60	2202 (28.3%)
>60	5590 (71.7%)
Sex	
Female	3627 (46.5%)
Male	4165 (53.5%)
Race	
White	6441 (82.7%)
Black	721 (9.3%)
Others	630 (8.0%)
Site	
Upper lobe	4213 (54.1%)
Lower lobe	2731 (35.0%)
Main bronchus	148 (1.9%)
Middle lobe	367 (4.7%)
Overlapping	333 (4.3%)
Grade	
Ⅰ/Ⅱ	3988 (51.2%)
Ⅲ/Ⅳ	3804 (48.8%)
Histology	
Adenocarcinoma	5117 (65.7%)
Squamous cell carcinoma	2675 (34.3%)
T stage	
T1	2028 (26.0%)
T2	4411 (56.6%)
T3	457 (5.9%)
T4	896 (11.5%)

**Table 2:** Description of baseline patient characteristics based on lymph node ratio.

	LNR1 (N=3585)	LNR2 (N=3397)	LNR3 (N=810)	P
Age				0.426
≤60	1039 (29.0%)	939 (27.6%)	224 (27.7%)	
>60	2546 (71.0%)	2458 (72.4%)	586 (72.3%)	
Sex				<0.001
Female	1581 (44.1%)	1639 (48.2%)	407 (50.2%)	
Male	2004 (55.9%)	1758 (51.8%)	403 (49.8%)	
Race				0.010
White	3021 (84.3%)	2761 (81.3%)	659 (81.4%)	
Black	296 (8.3%)	338 (9.9%)	87 (10.7%)	
Others	268 (7.4%)	298 (8.8%)	64 (7.9%)	
Site				<0.001
Upper lobe	1994 (55.6%)	1814 (53.4%)	405 (50.0%)	
Lower lobe	1228 (34.3%)	1221 (35.9%)	282 (34.8%)	
Main bronchus	83 (2.3%)	55 (1.6%)	10 (1.2%)	
Middle lobe	134 (3.7%)	173 (5.2%)	60 (7.5%)	
Overlapping	146 (4.1%)	134 (3.9%)	53 (6.5%)	
Grade				<0.001
Ⅰ/Ⅱ	1897 (52.9%)	1746 (51.4%)	345 (42.6%)	
Ⅲ/Ⅳ	1688 (47.1%)	1651 (48.6%)	465 (57.4%)	
Histology				<0.001
Adenocarcinoma	2141 (59.7%)	2354 (69.3%)	622 (76.8%)	
Squamous cell carcinoma	1444 (40.3%)	1043 (30.7%)	188 (23.2%)	
T stage				<0.001
T1	898 (25.1%)	926 (27.3%)	204 (25.2%)	
T2	2111 (58.9%)	1940 (57.1%)	360 (44.4%)	
T3	238 (6.6%)	172 (5.1%)	47 (5.8%)	
T4	338 (9.4%)	359 (10.6%)	199 (24.6%)	

LNR: lymph node ratio.

**Table 3:** Description of baseline patient characteristics based on pN stage.

	N1 (N=4999)	N2 (N=2660)	N3 (N=133)	P
Age				0.122
≤60	1424 (28.5%)	731 (27.5%)	47 (35.3%)	
>60	3575 (71.5%)	1929 (72.5%)	86 (64.7%)	
Sex				<0.001
Female	2252 (45.0%)	1319 (49.6%)	56 (42.1%)	
Male	2747 (55.0%)	1341 (50.4%)	77 (57.9%)	
Race				0.203
White	4170 (83.4%)	2161 (81.2%)	110 (82.7%)	
Black	445 (8.9%)	263 (9.9%)	13 (9.8%)	
Others	384 (7.7%)	236 (8.9%)	10 (7.5%)	
Site				0.001
Upper lobe	2685 (53.7%)	1458 (54.8%)	70 (52.6%)	
Lower lobe	1765 (35.3%)	925 (34.8%)	41 (30.8%)	
Main bronchus	105 (2.1%)	40 (1.5%)	3 (2.3%)	
Middle lobe	241 (4.8%)	123 (4.6%)	3 (2.3%)	
Overlapping	203 (4.1%)	114 (4.3%)	16 (12.0%)	
Grade				<0.001
Ⅰ/Ⅱ	2613 (52.3%)	1330 (50.0%)	45 (33.8%)	
Ⅲ/Ⅳ	2386 (47.7%)	1330 (50.0%)	88 (66.2%)	
Histology				<0.001
Adenocarcinoma	3110 (62.2%)	1917 (72.1%)	90 (67.7%)	
Squamous cell carcinoma	1889 (37.8%)	743 (27.9%)	43 (32.3%)	
T stage				<0.001
T1	1317 (26.3%)	673 (25.3%)	38 (28.6%)	
T2	2906 (58.2%)	1462 (55.0%)	43 (32.3%)	
T3	296 (5.9%)	155 (5.8%)	6 (4.5%)	
T4	480 (9.6%)	370 (13.9%)	46 (34.6%)	

**Table 4:** Cancer-specific survival and overall survival based on LNR and pN stages.

	LNR			N		
	LNR1	LNR2	LNR3	N1	N2	N3
Cancer-Specific Survival						
No. of events	1351	1738	569	2039	1514	105
Median (months)	71	41	17	62	33	14
HR (95% CI)	reference	1.46 (1.36-1.57)	2.85 (2.58-3.15)	reference	1.61 (1.51-1.73)	3.21 (2.63-3.92)
P value$	<0.001			<0.001		
Overall Survival						
No. of events	1750	2067	651	2599	1757	112
Median (months)	50	35	16	46	29	13
HR (95% CI)	reference	1.36 (1.27-1.45)	2.60 (2.37-2.85)	reference	1.50 (1.41-1.60)	2.81 (2.33-3.41)
P value$	<0.001			<0.001		

LNR: lymph node ratio. HR: hazard ratio. CI: confidence interval. $: P values were obtained with the use of the log-rank test for overall comparisons among three groups. Pairwise comparisons among groups are also statistically significant with the adjustment of Benjamini-Hochberg method.

**Table 5:** Comparison of predictive performance for cancer-specific survival and overall survival based on LNR, pN, and r-pN stages.

	Cancer-specific survival		Overall Survival	
	AIC^$^	Harrell's C-index^&^	AIC^$^	Harrell's C-index^&^
LNR	59919	0.648	72957	0.642
pN	60040	0.640	73100	0.633
r-pN	59814	0.656	72872	0.648

LNR: lymph node ratio. r-pN: revised pN. AIC: akaike information criterion. $: The lower the AIC value is, the better the calibration. &: The higher the Harrell's C-index is, the better the discrimination.

## Abbreviations

NSCLC: non-small cell lung cancer
NCCN: National Comprehensive Cancer Network
IASLC: International Association for the Study of Lung Cancer
ESTS: European Society of Thoracic Surgery
LNR: lymph node ratio
SEER: Surveillance, Epidemiology, and End Results cancer databases
TNM: tumor-node-metastasis
AJCC: American Joint Committee on Cancer
CSS: cancer-specific survival
OS: overall survival
C-index: the Harrell's concordance index
AIC: akaike information criterion
HR: hazard ratio
CI: confidence interval
